# Cardiac Fibroblasts Regulate Sympathetic Nerve Sprouting and Neurocardiac Synapse Stability

**DOI:** 10.1371/journal.pone.0079068

**Published:** 2013-11-14

**Authors:** Céline Mias, Christelle Coatrieux, Colette Denis, Gaël Genet, Marie-Hélène Seguelas, Nathalie Laplace, Charlotte Rouzaud-Laborde, Denis Calise, Angelo Parini, Daniel Cussac, Atul Pathak, Jean-Michel Sénard, Céline Galés

**Affiliations:** 1 Institut des Maladies Métaboliques et Cardiovasculaires, Institut National de la Santé et de la Recherche Médicale, U1048, Université Toulouse III Paul Sabatier, Toulouse, France; 2 Department of Microsurgery, Institut National de la Santé et de la Recherche Médicale UMS006, Toulouse, France; 3 Department of Pharmacology, Centre Hospitalier Universitaire de Toulouse, Toulouse, France; Georgia Regents University, United States of America

## Abstract

Sympathetic nervous system (SNS) plays a key role in cardiac homeostasis and its deregulations always associate with bad clinical outcomes. To date, little is known about molecular mechanisms regulating cardiac sympathetic innervation. The aim of the study was to determine the role of fibroblasts in heart sympathetic innervation. RT-qPCR and western-blots analysis performed in cardiomyocytes and fibroblasts isolated from healthy adult rat hearts revealed that Pro-Nerve growth factor (NGF) and pro-differentiating mature NGF were the most abundant neurotrophins expressed in cardiac fibroblasts while barely detectable in cardiomyocytes. When cultured with cardiac fibroblasts or fibroblast-conditioned medium, PC12 cells differentiated into/sympathetic-like neurons expressing axonal marker Tau-1 at neurites in contact with cardiomyocytes. This was prevented by anti-NGF blocking antibodies suggesting a paracrine action of NGF secreted by fibroblasts. When co-cultured with cardiomyocytes to mimic neurocardiac synapse, differentiated PC12 cells exhibited enhanced norepinephrine secretion as quantified by HPLC compared to PC12 cultured alone while co-culture with fibroblasts had no effect. However, when supplemented to PC12-cardiomyocytes co-culture, fibroblasts allowed long-term survival of the neurocardiac synapse. Activated fibroblasts (myofibroblasts) isolated from myocardial infarction rat hearts exhibited significantly higher mature NGF expression than normal fibroblasts and also promoted PC12 cells differentiation. Within the ischemic area lacking cardiomyocytes and neurocardiac synapses, tyrosine hydroxylase immunoreactivity was increased and associated with local anarchical and immature sympathetic hyperinnervation but tissue norepinephrine content was similar to that of normal cardiac tissue, suggesting depressed sympathetic function. Collectively, these findings demonstrate for the first time that fibroblasts are essential for the setting of cardiac sympathetic innervation and neurocardiac synapse stability. They also suggest that neurocardiac synapse functionality relies on a triptych with tight interaction between sympathetic nerve endings, cardiomyocytes and fibroblasts. Deregulations of this triptych may be involved in pathophysiology of cardiac diseases.

## Introduction

Sympathetic nervous system (SNS) plays a critical role in the maintenance of cardiovascular homeostasis by regulating intrinsic heart functions. Indeed, cardiac sympathetic nerves are extensively sprouted throughout the cardiac tissue and their stimulation promotes norepinephrine (NE) secretion which in turn contributes to the modulation of heart rate, conduction, velocity, contractility [1] but also exert trophic action on cardiac tissue. Thus, either an increase or a decrease in sympathetic activity directly impact cardiac tissue remodelling and heart functions. For instance, increased NE in transgenic mice model was directly associated with development of left ventricular hypertrophy and heart failure [2], [3]. Similar cardiac remodelling has been reported in patients with primary autonomic failure [4]. Beside SNS activity, physical innervation *per se* also contributes to deleterious cardiac effects. For instance, a number of human pathologies associated with either cardiac sympathetic hyperinnervation such as myocardial infarction (MI) [5] or conversely hypoinnervation such as diabetic or α-synuclein-associated postganglionic autonomic neuropathies [6], [7] are associated with increased cardiac morbi-mortality [8], [9]. All these data strongly argue for a crucial role of SNS innervation in the heart, thus reinforcing the need to improve our knowledge on molecular and cellular mechanisms contributing to the regulation of cardiac SNS innervation.

Among the large number of neurotrophic factors that have been shown to participate in the development, maturation and differentiation of cardiac sympathetic nerves [10], the neurotrophin family was more recently assigned an essential role in cardiovascular functions [11]. Neurotrophins play an important role in the regulation of the cardiac SNS, acting as trophic, survival factors but also as regulators of axonal arborization, with nerve growth factor (NGF) being the most extensively studied member of this family. Indeed, NGF is the major trophic factor for sympathetic nerves supporting not only their growth but also their survival and differentiation and promoting cardiac nerve outgrowth during the development and in pathological conditions [12], [13], [14]. Moreover, recent studies reported both *in vitro* or *in vivo* evidences for beneficial actions of NGF on cardiomyocytes in normal and pathological heart including pro-survival and anti-apoptotic effects [11], [15], [16]. The level of NGF in the target organ is also directly correlated to sympathetic innervation density [17]. Thus, in MI, a persistent up-regulation of NGF expression is observed within the ischemic area of infarcted hearts, underlying its implication in post-infarction nerve sprouting [18], [19]. Furthermore, NGF blocking antibodies prevented the outgrowth of sympathetic ganglia promoted by peri-infarct cell explants [20]. Various cardiac non-neuronal cells, such as cardiomyocytes, macrophages and myofibroblasts, have been shown to participate in NGF secretion in the cardiac tissue but in pathophysiological situations [20], [21], [22], [23], [24]. However, direct assessment of NGF secretion by the individual cardiac cells and in physiological conditions has never been evaluated.

In the present study, we directly evaluated neurotrophins expression and secretion in isolated resident adult cardiomyocytes and fibroblasts. We found that, in physiological conditions, fibroblasts, but not cardiomyocytes, produced high level of NGF, thus allowing PC12 cell sympathetic–like neuronal differentiation and “long term” stabilization but also functionality of the connection established between sympathetic neurones and cardiomyocytes. Similarly, activated myofibroblasts isolated from the cardiac ischemic area following myocardial infarction, overexpressed NGF correlating with sympathetic anarchical hyperinnervation in the tissue scar. However, in the absence of cardiomyocytes in the scar, hyperinnervation harbors decreased function. Altogether, these results highlight for the first time the essential triptych association between cardiomyocytes, sympathetic nerve endings and fibroblasts in the functionality of the neurocardiac synapse. They also suggest the putative role of cardiac myofibroblasts in the SNS deregulations in ischemic cardiac diseases.

## Materials and Methods

### Ethics Statement

Rat experiments were approved and performed according to the guidelines of the Ethics and Animal Safety Committee of INSERM Toulouse (agreement number: C3155507).

### Isolation and primary culture of adult cardiac fibroblasts and myofibroblasts

Adult cardiac fibroblasts (Fb) were isolated from ventricles of 2 months old Lewis rats. Ventricles were minced and digested in 0.05% collagenase, type IV (Worthington, NJ, USA), 0.5 mg/ml pancreatine, 1 mg/ml BSA solution at 37°C every 10 min until entire tissue digestion. Then, cells were plated on dishes in DMEM/F12 medium supplemented with 1% penicillin/streptomycin and 10% foetal bovine serum 37°C and 5% CO_2_. After 2 hours during which CFs have adhered to the dishes, non adherent cells were removed and culture medium was refreshed. All animal experiments were performed in accordance with the European Community guidelines for the use of experimental animals and national law. All experiments were performed on cells at passage 0. Cardiac fibroblasts were identified by positive staining for vimentin and negative for von Willebrand factor and troponin I. For myofibroblast primary culture, cells were isolated from the ischemic area of infarcted hearts and followed the same procedure. The ischemic area was carefully extracted by visually excluding the non-necrotic region. Cardiac myofibroblasts were identified by positive staining for alpha-smooth muscle actin and negative for von Willebrand factor and troponin I. To obtain Fibroblasts-conditioned medium, cardiac resident fibroblasts were isolated from rat hearts and cultured in 100 mm Petri dishes up to 80% confluence (4–5 days culture) in their specific culture medium (DMEM/F12, 1% penicillin/streptomycin, 10% foetal bovine serum) which was then collected as “fibroblast-conditioned medium” and stored at −80°C until use.

### Isolation and primary culture of adult rat ventricular cardiomyocytes

Left ventricular cardiomyocytes were isolated from adult rat hearts using standard procedures. Briefly, isolated heart was mounted on a Langendorf apparatus and perfused for 4 minutes at 3 ml/minutes with perfusion buffer containing: NaCl 113 mM, KCl 4.7 mM, KH_2_PO_4_ 0.6 mM, Na2HPO4 0.6 mM, MgSO_4_ 1.2 mM, NaHCO_3_ 12 mM, KHCO_3_ 10 mM, taurine 30 mM, glucose 5.5 mM, 2,3-butanedione monoxime 10 mM and HEPES 10 mM (pH 7.4). After the washing step, heart was perfused during 3 minutes with decalcifying solution (perfusion buffer complemented with EGTA 0.1 mM) and, then perfused during 10 minutes with digestion buffer (perfusion buffer complemented with 2.2 mg/ml type II collagenase (Worthington) and 12.5 µM CaCl_2_). Once enzymatic digestion completed, heart was removed from the Langendorf system, the atria were removed and the ventricles cut into small pieces. After filtration on a 200 µm nylon filter, the cardiomyocytes pellet was suspended in the resuspension medium (perfusion buffer, 5% FBS and 12,5 µM CaCl_2_).

### PC12 cell culture and differentiation

Rat pheochromocytoma PC12 cells were purchased from ECACC (European Collection of Cell Cultures - Catalog number: 88022401). Cells were cultured routinely with RPMI 1640 medium containing 10% heat-inactivated horse serum, 5% heat-inactivated foetal bovine serum (FBS) and 1% penicillin/streptomycin at 37°C in a 95% humidified incubator with 5% CO_2_. Under these conditions the cells grew mainly in suspension. To induce PC12 neurites-differentiation, cells were seeded in poly-L-lysine (1 mg/ml; Sigma) pre-coated culture 6-well plates at a density of 25×10^3^ per well and cultured in the specific differentiating medium (RPMI 1640, 1% horse serum, 1% penicillin/streptomycin, supplemented with 70 ng/ml NGF (2.5 S, Invitrogen, France)). Differentiation is complete after 6–8 days of NGF treatment. For NGF blocking experiments, PC12 cells were cultured in differentiation medium or fibroblast-conditioned medium (See above) in the presence of an anti-NGF 2.5S antibody (1/10000^e^, Sigma-Aldrich, USA) for 48 hours.

### PC12 cell culture with cardiac fibroblasts and/or cardiomyocytes

For PC12/Fb co-culture experiments, PC12 cells were seeded at equal density (25×10^3^ cells/well) in poly-L-lysine (1 mg/ml; Sigma) pre-coated culture 6-well plates and cultured over night in PC12 complete medium (RPMI 1640, 10% heat-inactivated horse serum, 5% heat-inactivated foetal bovine serum (FBS), 1% penicillin/streptomycin) to ensure cell adhesion. The next day, immediately after purification from rat hearts, resident fibroblasts were seeded (1.25×10^5^ cells/well) directly with PC12 adhesive cells and the co-culture was cultured in complete fibroblast medium or PC12 adhesive cells alone were cultured in the presence of fibroblast-conditioned medium (See above).

For PC12/Fb/CM tri-culture experiments, PC12 were first differentiated in 6-well plates in the presence of NGF during 8 days as indicated above. Then, immediately after purification from rat hearts, resident fibroblasts (or myofibroblasts from cardiac ischemic area) were seeded (1.25×10^5^ cells/well) directly with pre-differentiated-PC12 and the co-culture was then cultured in fibroblast complete medium. When considering the tri-culture (PC12-Fb+CM), fibroblasts adhesion was first ensure during 2 hours in fibroblast complete medium in the presence of pre-differentiated PC12 and isolated cardiomyocytes (10^4^ cells/well) were further added to the PC12-Fb co-culture and cultured in fibroblast complete medium.

### Immunocytochemistry

PC12 cells cultured alone or in presence of cardiac fibroblasts were fixed with 4% paraformaldehyde solution for 20 minutes at room temperature (RT). The cells were permeabilized with 0.1% Triton-X100 for 20 minutes at RT and blocked in 5% BSA/PBS for 1 hour at RT. For co-immunostaining, the cells were incubated with mouse monoclonal anti-Tau-1 antibody (1∶200, overnight, 4°C; Chemicon, Millipore, France), and with Alexa-fluor® 488 goat anti-mouse antibody (dilution 1∶200, 1 hour, room temperature; Invitrogen, France). Then, the cells were washed and blocked with PBS/5% BSA for 1 hour at RT and incubated with rabbit polyclonal anti-microtubule-associated protein 2 (MAP-2) antibody (dilution 1∶500, overnight, 4°C; Chemicon, Millipore, France), and with Texas-red® 488 goat anti-rabbit antibody (dilution 1∶200, 1 hour, room temperature; Invitrogen, France). Fluorescence images were acquired with an Axio Observer Z.1m inverted microscope (Carl Zeiss Jena, Germany). Co-localizations were assessed by overlay using the Zeiss microscopy software AxioVision Rel. 4.8.

### Neurite quantification

To measure induction of neurite outgrowth in PC12, optical microscopic images were taken 48 and 96 hours after co-culture with cardiac (myo)fibroblasts and/or cardiomyocytes. Only neurites exceeding more than two PC12 cell-body diameters were counted. Graphs represented the percentage of differentiated PC12 cells in each culture condition.

### Total RNA isolation and real time quantitative RT-PCR

Extraction of total RNA from isolated adult CMs or fibroblasts was performed using QIAGEN RNeasy Mini Kit (QIAGEN SA, France). First-strand cDNA was synthesized using the superscript II RT-PCR system (Invitrogen) with random hexamers. Negative controls without reverse transcriptase were made to verify the absence of genomic DNA contamination. Real-time PCR was performed in an ABI 7500 Fast (Applied Biosystems,) in 96-well plates. Fifteen nanograms of cDNA from RT reaction were then mixed with MesaGreen qPCR MasterMix Plus (Eurogentec, France) and the primers (Eurogentec, Belgium) listed in Table 1. Results were expressed relatively to the geometrical mean of the two most stable housekeeping genes GAPDH (Glyceraldehyde 3-Phosphate Dehydrogenase) and HPRT 1 (Hypoxanthine-guanine phosphoribosyltransferase-1). Relative expression was calculated by the comparative Ct method [25].

**Table 1:** Primer sets used in RT-PCR experiments.

Gene Name	Primer sequence
**NGF**	F: 5′-GGGCCCAATAAAGGCTTTGC-3′
	R: 5′- GTTCTGCCTGTACGCCGATC-3′
**NT3**	F: 5′-CTCTTCATGTCGACGTCCC-3′
	R: 5′-CGGAGATAAGCAAGAAATATCA-3′
**NT4**	F: 5′- AGGTGGTTGCCCCCTCCTT-3′
	R: 5′- GCACACCTGTCAACAGCACCT-3′
**BDNF**	F: 5′-CATCCAGCGCACCTCTTTAGG-3′
	R: 5′-CATCCAGCGCACCTCTTTAGG-3′
**CNTF**	F: 5′-CGTTCTATCTGGCTAGCAAG-3′
	R: 5′-CTGGTACACCATCCACTGAG-3′
**GAPDH**	F: 5′-AGGTCGGTGTGAACGGATTTG-3′
	R: 5′-ATGTAGACCATGTAGTTGAGGTC-3′
**HPRT1**	F: 5′-TGACACTGGTAAAACAATGCAGAC-3′
	R: 5′-GAGAGGTCCTTTTCACCAGCAAG-3′

### Western-Blotting

For protein analysis from fibroblasts and PC12 cells, cells were directly lysed in RIPA buffer (25 mM Tris-HCl pH 7.6, 150 mM NaCl, 1% NP-40, 1% NaDoc, 0.1% SDS) in the presence of protease inhibitors cocktail (Roche). Protein concentration of extracts was determined by the Bradford method (Bio-Rad) and equal amounts of proteins (50 µg) were subjected directly to SDS-PAGE and transferred to nitrocellulose membranes (Millipore). Proteins were detected with rabbit anti-NGF 2.5S primary antibody (Sigma, USA, dilution 1∶1000) followed by Horse Radish Peroxydase-conjugated anti-rabbit secondary antibodies (dilution 1∶10000; Santa Cruz Biotechnology, USA,) using enhanced chemoluminescence detection reagent (GE Healthcare). Protein quantification was obtained by densitometric analysis using ImageQuant 5.2 software.

### Immunocytochemistry

After 48 and 96 hours of culture, cells were fixed with 4% paraformaldehyde in PBS for 15 minutes, permeabilized for 10 minutes with 0.3% Triton X-100 in blocking buffer (PBS/0.2% BSA) and incubated for 30 minutes with both a rabbit anti-tyrosine hydroxylase antibody (Chemicon, France) and a goat anti-vimentin antibody (Santa Cruz biotechonology, France), at a dilution of 1∶100 in blocking buffer. Immunoreactivity was revealed using Oregon green 488® conjugated secondary goat anti-rabbit antibody and Alexa-fluor® 568 conjugated secondary rat anti-goat antibody (Molecular Probes, France), at a dilution of 1∶1000 in blocking buffer. Before microscopic analysis, DAPI solution was added to dishes (10 minutes, room temperature). Images were acquired using a Zeiss observer Z.1 microscope and analyzed using Axiovision Rel 4.7 software.

### Norepinephrine concentration quantification

Norepinephrine (NE) was assayed by high pressure liquid chromatography using electrochemical (amperometric) detection and 3,4-dihydrobenzylamine as an internal standard as previously described [26]. For experiments using PC12 cells, NE concentration was measured in PC12 differentiated in the presence of NGF during 8 days as indicated above. To then measure NE secretion, culture medium was removed and replaced by Hank's Buffered Salt Solution (HBSS) for 10 minutes in the incubator. HBSS was then collected, centrifuged and frozen at −80°C until analysis. Cell number was determined and final result was expressed in pg/10 min/10^3^ cells. For the evaluation of NE concentration in heart tissue from rats, heart samples were rapidly collected after sacrifice and immediately homogenized in ice-cold 0.4N perchloric acid. After centrifugation, extraction of catecholamines was done on alumina in a buffered medium (pH 8.6) containing 0.36% EDTA, washed several times with deionized water, and eluted in 200 µL of 0.1 N perchloric acid. Tissue content in NE is expressed in pg/mg of protein.

### Myocardial infarction rat model

Lewis male rats (180–200 g, Harlan, France) were anesthetized by a mix of isoflurane/oxygen inhalation (3/97). After left lateral thoracotomy, heart was accessed through the fourth intercostals space. Interventricular artery was ligated with a 6-0 polyester suture. Sham-operated animals were subjected to similar surgical procedure without coronary artery ligation.

### Histology and Immunohistochemistry

Control or infarcted hearts were removed and collected 15 days after surgery. Paraffin embedded-sections (6 µm) of hearts were stained with Masson's trichrome solution (collagen I and III coloration) using standard methods. For myofibroblasts detection, heart sections were successively incubated with mouse monoclonal anti-α-smooth muscle actin (α -SMA) antibody (dilution 1∶100, 90 minutes, room temperature; Sigma Aldrich) and with Alexa-fluor® 568 goat anti-mouse antibody (dilution 1∶200, 1 hour, RT; Invitrogen). For sympathetic nerve detection, heart sections were successively incubated with mouse monoclonal anti-tyrosine hydroxylase (TH) antibody (dilution 1∶50, 90 minutes, RT; Chemicon) and with Alexa-fluor® 488 goat anti-mouse antibody (dilution 1∶200, 1 hour, RT; Invitrogen). For detection of immature neurons, heart sections were successively incubated with mouse monoclonal anti-polysialic acid-NCAM (PSA-NCAM) antibody (dilution 1∶100, 1 hour, overnight, 4°C, Chemicon) and with Alexa-fluorR 568 goat anti-mouse antibody (dilution 1∶100, 1 hour, RT, Invitrogen). Quantification of histological sections were performed with computerized image analysis carried out on 3 fields of control or infarcted left ventricles (α-SMA and TH immunostaining); or on whole cardiac sections (Masson's trichrome coloration) of three different histological preparations obtained from each animal.

### Statistical analysis

Data are represented as mean +/−SEM. Statistical comparison of the data was performed using t-test for comparison between two groups or one-way ANOVA and post-hoc Tukey's test for comparison of more than two groups. A value of *p*<0.05 was considered statistically significant.

## Results

### Neurotrophic factors gene/protein expression in adult cardiomyocytes and cardiac fibroblasts

**Figure 1:**
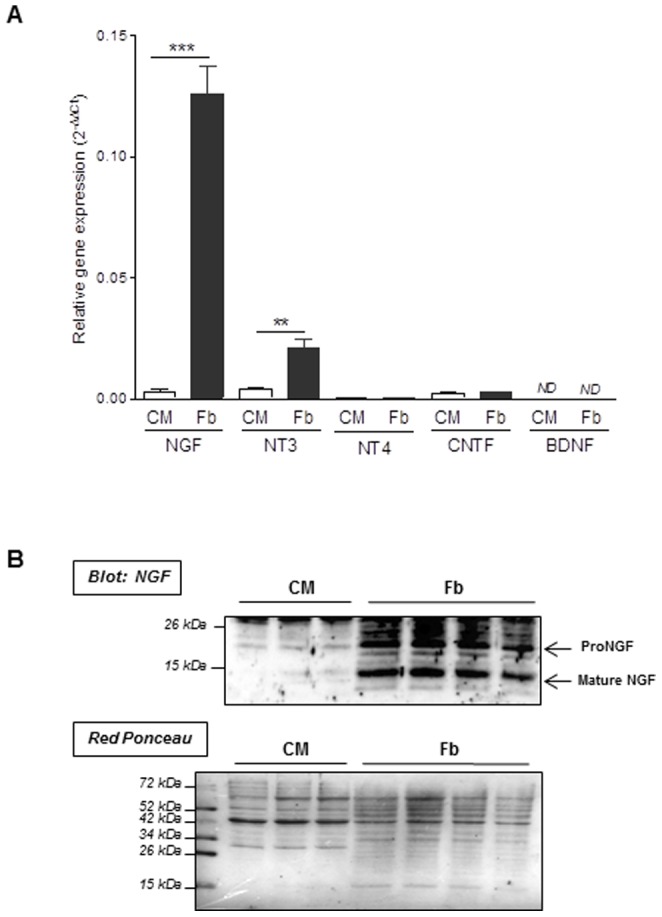
Neurotrophic factor gene expression profiling in isolated adult cardiomyocytes and cardiac fibroblasts. **A** NGF, NT3, NT4, CNTF and BDNF gene expression were measured by real time qPCR using mRNA extracted from cardiomyocytes (CM) and cardiac fibroblasts (Fb) isolated from adult control heart. Values represent the mean ±SEM. (***, *p<0.001* vs. CM; **p<0.001 vs. CM; *ND*: no detectable; n = 3–5). **B** NGF protein expression was evaluated by Western-blot experiments on primary cultures of adult cardiomyocytes (CM) and adult cardiac fibroblasts (Fb). Lower panel: Ponceau Red staining to control for equal protein loading between CM and Fb samples used for NGF blotting (upper).

To compare neurotrophin gene expression profiling in cardiomyocytes and fibroblasts, NGF, NT3, NT4, CNTF and BDNF gene expression were measured by real time qPCR using mRNA extracted from adult cardiomyocytes or cardiac fibroblasts isolated from healthy rat hearts. Cardiomyocytes exhibited very low levels of all neurotrophins genes expression under physiological conditions. By opposition, only significant expression of NGF and NT3 transcripts was depicted in fibroblasts when compared to other neurotrophic factors with much higher levels of NGF gene expression than NT3. Thus, fibroblasts demonstrated significant higher levels of NGF and NT3 transcripts when compared to cardiomyocytes (Figure 1A). In agreement with qPCR gene profiling, western-blot experiments confirmed the overexpression of immature proNGF but also mature NGF proteins in cardiac fibroblasts isolated from control hearts by opposition to adult cardiomyocytes in which the two proteins were barely detectable (Figure 1B). Because NGF was markedly much highly expressed than NT3 by fibroblasts (Figure 1A) and since mature NGF is the main neurotrophin involved in sympathetic neurons differentiation, survival, and function (neurotransmitter production) [27], [28], we further focused our study on NGF.

### Effects of cardiac fibroblasts on PC12 cell axonal/neuritogenesis

**Figure 2:**
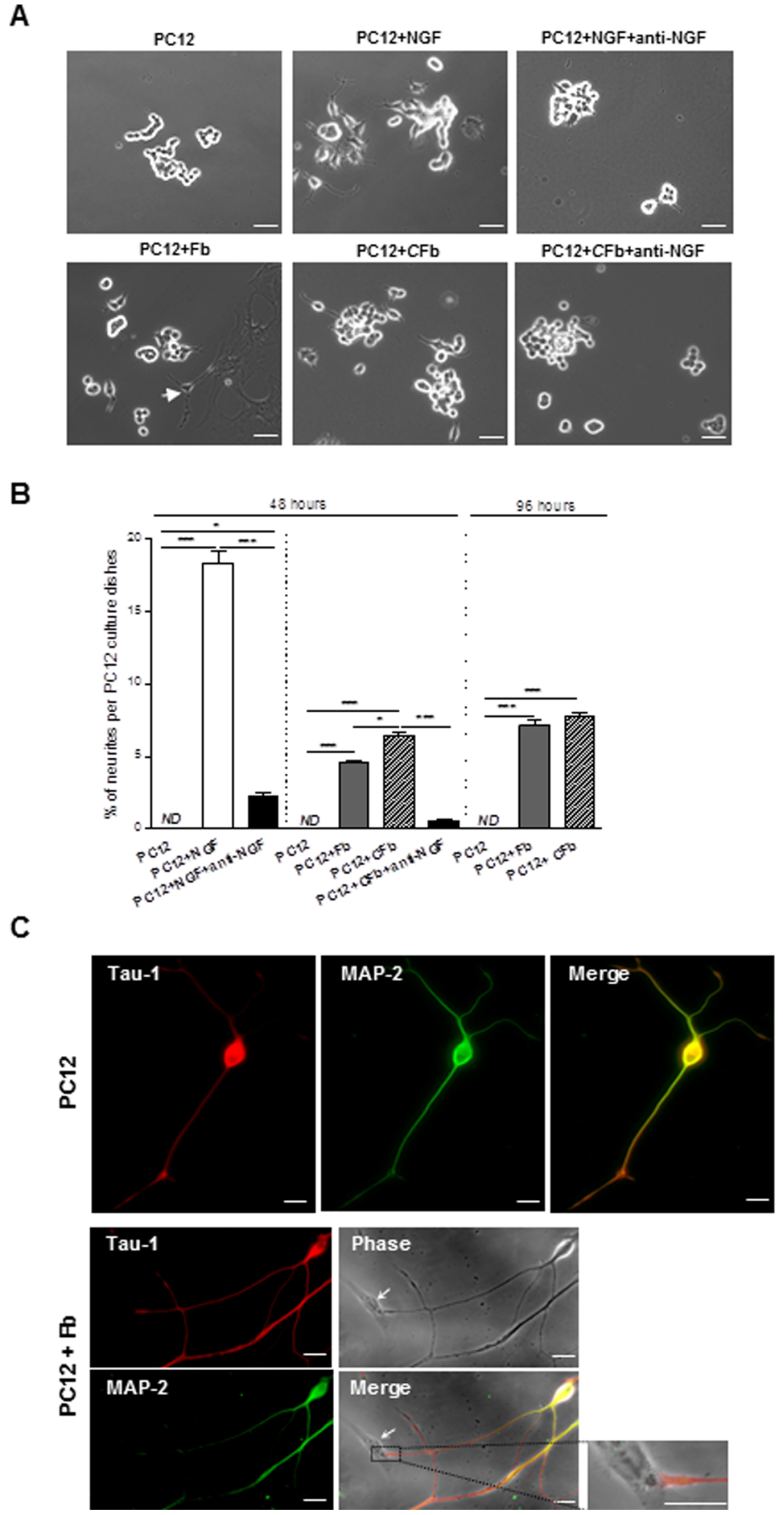
Cardiac fibroblasts promote PC12 cells axonal/neuritogenesis through NGF paracrine action. Representative images (**A**) and quantification (**B**) of PC12 cells cultured alone (PC12), PC12 treated with NGF (70 ng/ml) as a positive control for differentiated PC12 (P12+NGF), PC12 cultured in the presence of an anti-NGF antibody and treated with NGF (70 ng/ml) (PC12+NGF+anti-NGF), PC12 co-cultured with cardiac fibroblasts (PC12+Fb), PC12 cultured with cardiac fibroblast conditioned media (PC12+*C*Fb), PC12 co-cultured with cardiac fibroblast conditioned media in the presence of an anti-NGF antibody (PC12+*C*Fb+anti-NGF), 48 or 96 hours following supplementation. (n = 4–6 per group). Scale bars  = 50 µm. **C** Immunofluorescent co-staining for Tau-1 (axonal marker) and MAP-2 (dendritic marker) in PC12 cells cultured alone (upper) or co-cultured with cardiac fibroblasts (lower) (arrow: fibroblast). Scale bar  = 20 µm.

Based on these results, we next investigated the role of cardiac fibroblasts on neuritogenesis by measuring neurite outgrowth in PC12 cell cultures, which generally differentiate into neuron-like phenotypes upon NGF stimulation [29]. As shown in Figure 2A, neurite extensions were not detectable in PC12 cells when cultivated under classical conditions. However, when cells were serum starved and cultured in the presence of NGF for 48 hours, they developed a significant increase in neurites (18.0±0.8%) (Figure 2A, and 2B, PC12+NGF). NGF-mediated PC12 differentiation was almost completely suppressed upon anti-NGF blocking antibody treatment, demonstrating the anti-NGF efficacy (Figure 2A, and 2B; PC12+NGF+anti-NGF). Interestingly, co-culture with cardiac fibroblasts resulted in a significant increase of neurites outgrowth in PC12 cells, 48 hours (4.6±0.1%) and 96 hours (7.1±0.4%) following fibroblasts supplementation (Figure 2A, and 2B; PC12+Fb). Similar results were obtained when using cardiac fibroblast conditioned media (6.4±0.2% neurites outgrowth at 48 hours and 7.8±0.1% at 96 hours) (Figure 2A, and 2B; PC12+*C*Fb). Pretreatment with anti-NGF blocking antibody significantly decreased PC12 differentiation by fibroblasts conditioned media by about 90% (Figure 2A, and 2B; PC12+*C*Fb+anti-NGF), thus indicating that cardiac fibroblasts mainly promoted PC12 neuritogenesis through a specific and paracrin action of NGF. Interestingly, we noticed that NGF treatment of PC12 generated anarchical neurite extensions while organized neurites were observed in PC12-fibroblasts co-culture with neurite tips establishing synaptic contacts with fibroblasts. In agreement with previous results from Jeon *et al* [30], NGF promoted the differentiation of axonal neurite of PC12 cells as evidenced by the high concentration of Tau-1 expression, a specific axonal marker, at the terminal tips of neurite when compared to MAP-2 dendritic marker which was more concentrated in the cell body and proximal neurites (Figure 2C, upper panels). Moreover, when PC12 cells were co-cultured in the presence of cardiac fibroblasts, we could notice highly enriched Tau-1 terminal tips of PC12-neurites physically interacting with fibroblasts (Figure 2C, lower panels), thus suggesting the existence of mature synapses.

### Impact of cardiac fibroblasts on neurocardiac synapse structure and functionality

**Figure 3:**
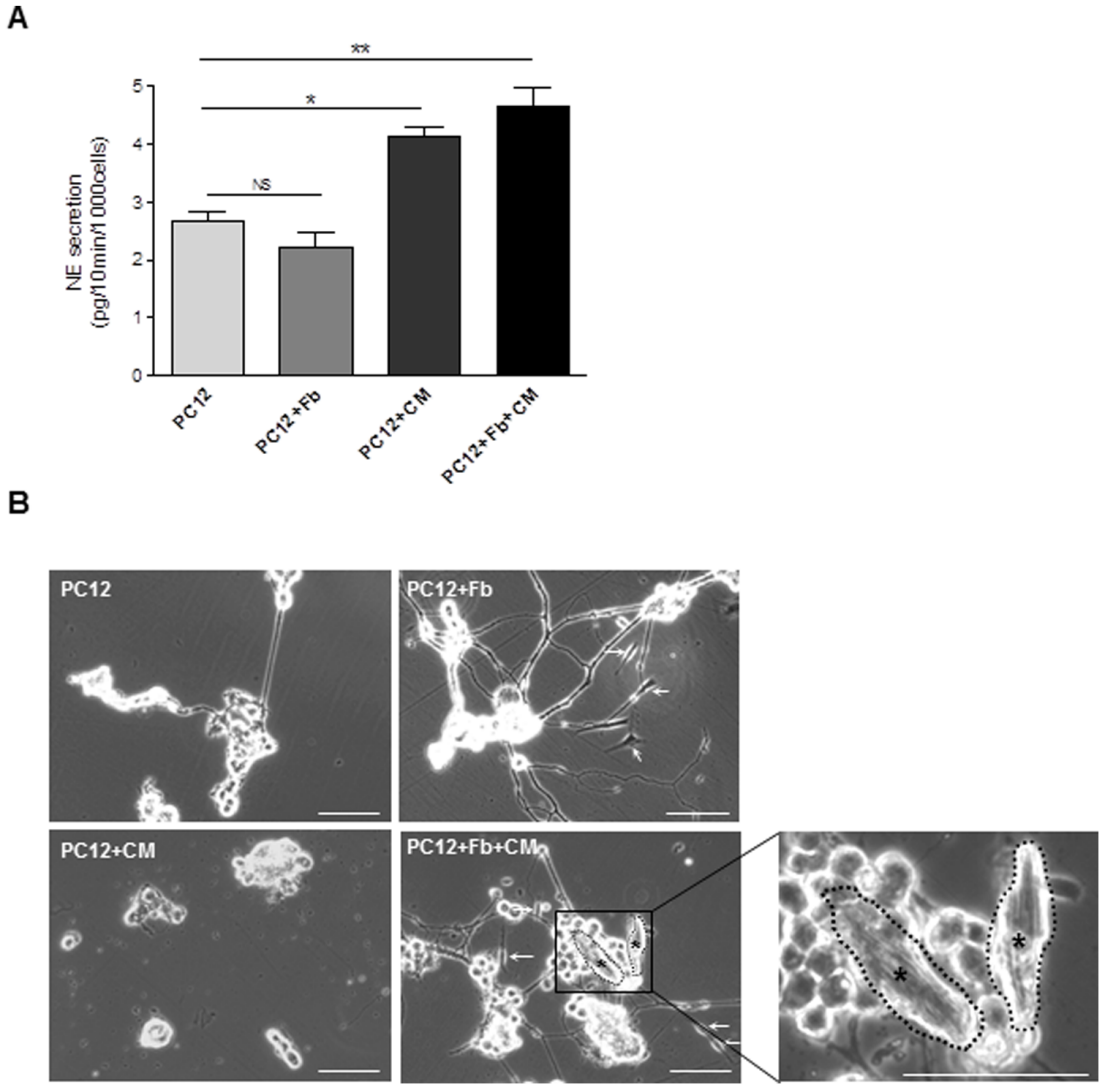
Impact of cardiac fibroblasts on neurocardiac synapse structure/functionality. (**A**) Neurocardiac synapse function: norepinephrine secretion by NGF-differentiated PC12 was quantified by HPLC in the culture medium under 48 hours different culture conditions: PC12 cultured alone (PC12), fibroblasts/PC12 co-culture (PC12+Fb), PC12/cardiomyocytes co-culture (PC12+CM) or PC12/cardiomyocytes/fibroblasts tri-culture (PC12+Fb+CM). *, *p<0.05* vs. PC12; **, *p<0.01* vs. PC12; NS: no statistical difference (n = 3–4). (**B**) Neurocardiac synapse structure: photomicrographs of PC12 cells cultured alone or PC12/CM co-culture show neurite degeneration when compared with PC12+Fb co-culture and PC12+Fb+CM tri-culture, after 3 days in culture. (arrows: fibroblasts, asterisks: cardiomyocytes). Scale bars  = 100 µm. In all experiments, PC12 neuritogenesis was promoted by 8-days incubation in the presence of NGF (See materials and methods).

To get further insight the consequences of NGF secretion by cardiac fibroblasts on sympathetic-like nerves mimicking sympathetic innervation in the heart, we next assessed its effect on the functionality of neuron-differentiated PC12 cells. For that purpose, we quantified norepinephrine (NE) secretion by chromatography in NGF-differentiated PC12 cells under different culture conditions: i/PC12 alone, ii/PC12-fibroblasts co-culture, iii/PC12-cardiomyocytes co-culture, or iv/PC12-cardiomyocytes-fibroblasts tri-culture. In all conditions, PC12 cells were first cultured during 8 days in neurone-differentiation medium (in the presence of NGF) and then switched to cardiac fibroblast culture conditions (without NGF addition) even when cultured in the presence of cardiomyocytes. NE concentration was measured in culture supernatant two days following co- or tri-culture of PC12 cells. As shown in Figure 3A, NGF-differentiated PC12 cells already secreted NE in the absence of neurites-cell contact, indicating that NE secretion is indeed possible in the absence of synapse. Interestingly, further addition of cardiac fibroblasts did not modify NE secretion (Figure 3A, PC12+Fb). By opposition, co-culture of PC12 cells in the presence of cardiomyocytes significantly stimulated the activity of PC12 by increasing their ability to secrete NE (Figure 3A, PC12+CM). This result demonstrated the ability of cardiomyocytes to establish a functional cell-cell contact with differentiated PC12 cells. It is noteworthy that the presence of PC12 influenced adult cardiomyocytes morphology in culture that kept their typical rod-shape while they rapidly underwent rounded shape when cultured alone (not shown). The addition of fibroblasts in the cardiomyocytes-PC12 co-culture did not modify the level of NE secretion by PC12 (Figure 3A, PC12+Fb+CM), thus confirming the incapability of fibroblasts to modulate NE secretion and thus neurocardiac synapse activity. However and interestingly, when cell cultures were extended over two days (3 days) in fibroblast medium culture conditions, we noticed a clear influence of fibroblasts on the stability of neurocardiac synapse architecture established between differentiated-PC12 nerves ending and cardiomyocytes (Figure 3B). Indeed, in the absence of fibroblasts, PC12 underwent rapid neurite degeneration (Figure 3B, PC12 and PC12+CM) and ensuing synapse disintegration (Figure 3B, PC12+CM) when compared to the “tri-culture” model (Figure 3B, PC12+Fb+CM) where several synapses could readily still be observed throughout the culture. PC12 neurites degeneration observed in the cardiac fibroblast culture conditions most likely related to the lack of NGF in the medium. By opposition, when cultured alone in poly-L-lysine pre-coated plates, cardiomyocytes were prone to rapid death 24 hours following isolation (not shown). Overall, these results indicate that cardiac fibroblasts, most probably through their ability to secrete NGF, are necessary to maintain the integrity of the neurocardiac synapse (nerves-cardiomyocytes) and play a key role in the “long-term” stabilization of the synapse architecture, thus allowing its sustained functionality.

### Effects of myofibroblasts on neuritogenesis and neurocardiac synapse functionality

**Figure 4:**
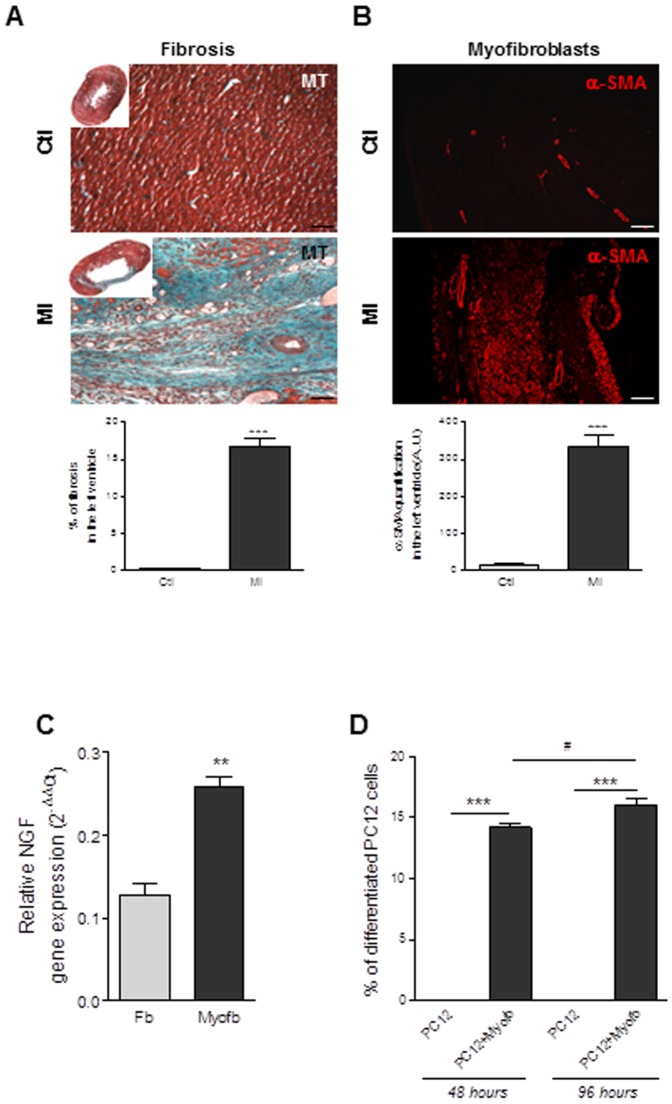
Myocardial infarction is associated with NGF over-expression by myofibroblasts which promotes PC12 cells axonal neuritogenesis. Photomicrographs and quantitative analysis of myocardial sections stained with Masson's trichrome (MT) (**A**), α-smooth muscle actin (α-SMA) (**B**). (**C**) Quantification of NGF gene expression in cardiac fibroblasts isolated from sham-operated rats (Fb) and in cardiac myofibroblasts isolated from the ischemic region of infarcted heart (Myofb). **, p<0.001 vs. Fb (n = 3–4 per rat group). (**D**) Neurite outgrowth quantification in PC12 cells, 48 and 96 hours after co-culture with myofibroblasts isolated from ischemic region of infarcted heart (PC12+Myofb). ***, p<0.001 vs. PC12, #, p<0.05 vs. PC12+Myofb 48 hours (n = 4).

Given the key role played by cardiac fibroblasts in both the stability and the function of the neurocardiac synapse together with the sympathetic dysfunction generally associated with fibrosis-associated cardiac diseases, we questioned about NGF secretion and function in activated fibroblasts (i.e. myofibroblasts). Thus, we isolated myofibroblasts from the ischemic area of rat ventricles fifteen days after myocardial infarction (MI). As expected [31], the ischemic area exhibited an important fibrotic scar characterized by the large absence of cardiomyocytes correlating with the presence of high number of α-smooth muscle actin positive cells indicative of myofibroblasts and associated with collagen (I and III types) deposition (Figure 4A and B). As shown in Figure 4C, NGF transcripts were markedly overexpressed in myofibroblasts when compared with normal fibroblasts. These results highly suggest the potential of myofibroblasts to secrete high levels of pro-differentiating NGF in response to MI even if we cannot exclude the participation of cardiomyocytes in the periphery of the scar area. Thus, we next investigated the ability of cardiac myofibroblasts to promote neurone-like differentiation of PC12, 48 hours or 96 hours after co-culture. As observed for normal resident cardiac fibroblasts, PC12 cells cultured with myofibroblasts displayed high number of newly-formed neurites while PC12 cells cultured alone displayed no extension (Figure 4D). PC12 neuritogenesis induced by myofibroblasts was significantly increased over time (96 versus 48 hours). Similar results were obtained when PC12 were cultured in cardiac myofibroblasts conditioned media (data not shown).

**Figure 5:**
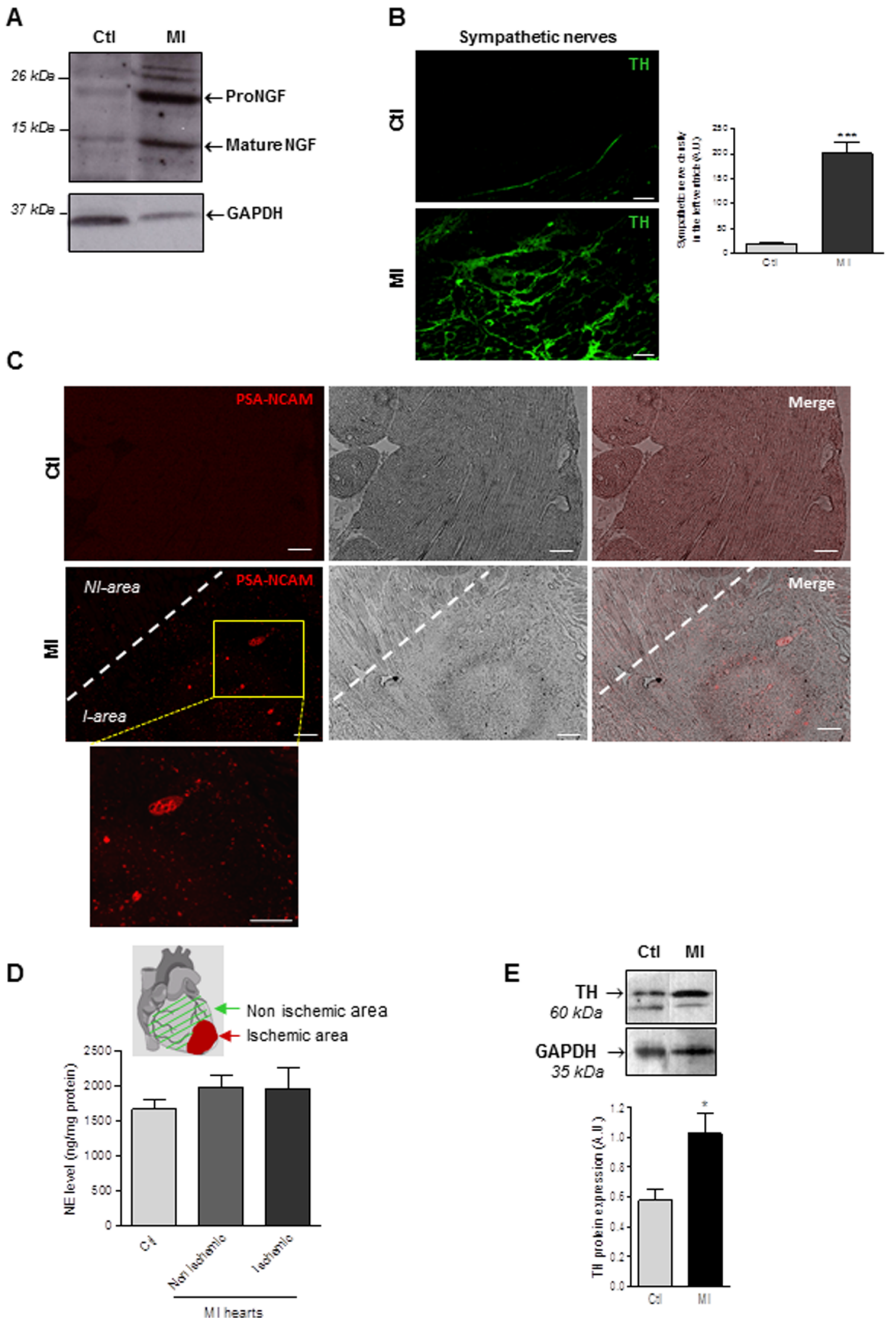
Myocardial infarction is associated with immature sympathetic heart hyperinnervation. (**A**) Representative Western-blot analysis of NGF expression in the ischemic area of infarcted area (MI), compared to sham-operated rats (Ctl). (**B**) Tyrosine hydroxylase-positive nerve fibers (TH) in sham-operated animals (Ctl) and within the ischemic area of infarcted heart 15 days following myocardial infarction (MI). Values are mean ±SEM. ***, *p*<0.001 vs. Ctl (n = 3–6 per rat lineage group). Scale bars  = 50 µm. (**C**) Immunofluorescent staining for PSA-NCAM (immature neurons marker) in myocardial sections of sham-operated animals (Ctl) and ischemic area (*I-area*) of infarcted hearts 15 days following myocardial infarction (MI). Scale bars  = 100 µm. (**D**) Cardiac tissue norepinephrine (NE) levels after myocardial infarction. Cardiac tissue norepinephrine was quantified by HPLC in sham-operated rats (Ctl) and in non-ischemic or ischemic areas of infarcted heart (MI), 15 days after myocardial infarction (n = 4–5 per rat group). (**E**) Representative and quantitative Western-blot analysis of cardiac tyrosine hydroxylase (TH) protein expression in sham-operated rats (Ctl) and in ischemic areas 15 days after myocardial infarction (MI). Values are means ±SEM. (n = 6 per rat group). *, p<0.05 vs. Ctl.

In order to verify if our *in vitro* findings could exhibit *in vivo* relevance, we then examined NGF expression in myofibroblast-enriched ischemic area. Western-blot experiments showed a significant overexpression of both immature proNGF and mature NGF in the infarction scar of rat hearts when compared to control tissue (Figure 5A). NGF overexpression in the scar tissue correlated with a marked increase in Tyrosine Hydroxylase (TH)-positive nerve density within the ischemic area when compared to normal tissue (Figure 5B) indicative of sympathetic hyperinnervation as previously described. It is noteworthy that sympathetic nerve fibers were totally disorganised in the ischemic region when compared to those observed in control hearts. However, sympathetic nerves within scar area exhibited a foetal non mature phenotype as indicated by the appearance of polysialylated neural cell adhesion molecule PSA-NCAM expression, a known marker of immature neurones (Figure 5C). These results indicate that sympathetic hyperinnervation mainly occurred in areas presenting large amount of myofibroblasts even in the absence of cardiomyocytes. Finally, we questioned about the functionality of the sympathetic hyperinnervation in the ischemic area by measuring tissue NE concentrations. Surprisingly, we observed that NE content within the myofibroblast-enriched ischemic area was similar to that of cardiac tissue regions only containing normal fibroblasts (Figure 5D), thus highly suggesting sympathetic nerves decreased functionality in the scar. Depressed nerve activity did not relate to a decrease in expression of TH, the rate-determining enzyme for catecholamine synthesis as previously reported [32], [33], since western-blots experiments indicated a significant increase in TH expression in the scar tissue when compared to the non ischemic area (Figure 5E), thus corroborating with our TH-immunohistochemistry (Figure 5B). Collectively, these results highlight the ability of myofibroblasts to promote NGF secretion and the development of immature and thus poorly functional sympathetic innervation within cardiac tissue.

## Discussion

In the present study, we highlighted for the first time the crucial role of cardiac fibroblasts in sympathetic neuritogenesis and long-term neurocardiac synapse stability.

Physiological cardiac tissue architecture is achieved by interaction of different cell types including fibroblasts, cardiomyocytes, macrophages and endothelial cells. Fibroblasts play a key role in the maintenance of adult cardiac tissue organization where they are responsible for cardiac cells framework, allowing their correct 3D organization and interconnections through extracellular matrix synthesis [34]. In addition to their role in cardiac tissue structure, fibroblasts also participate in the regulation of cardiomyocytes and endothelial cells function through the secretion of cytokines, chemokines and growth factors [35]. The ability of fibroblasts to secrete NGF has already been reported in different fibroblastic cell lines [36], [37] but, in this study, we provide for the first time clear evidence that isolated healthy cardiac fibroblasts secreted high level of NGF in physiological conditions. This highlights the importance of NGF in the paracrine communication of fibroblasts with cardiac neuronal cells. By contrast, adult cardiomyocytes exhibited very weak NGF expression in normal cardiac tissue, thus lessening their importance for NGF secretion in homeostatic conditions. However, the normal cellular patterns of NGF secretion may differ in pathological conditions. Up-regulation of NGF production by cardiomyocytes was observed in cardiac hypertrophy and upon endothelin-1 treatment [23]. Moreover, the implication of microvascular endothelial cells and immune cells in NGF secretion following cardiac ischemic diseases was also reported [20], [24], [38], [39].

**Figure 6:**
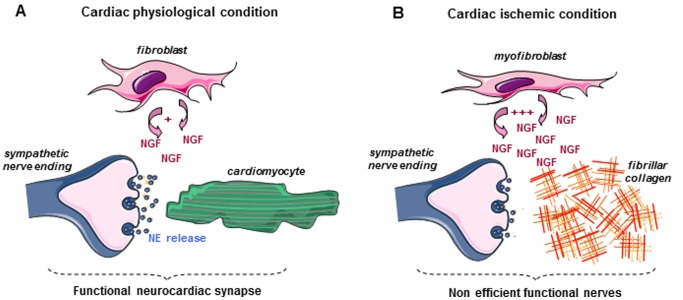
A proposed model for the tight cooperation between fibroblasts, cardiomyocytes and sympathetic nerves in the functionality of the neurocardiac synapse. **A** In physiological condition, the triptych association between cardiomyocytes, sympathetic nerve endings and fibroblasts allows a functional neurocardiac synapse. Indeed, fibroblasts stabilize nerves ending-cardiomyocytes physical interaction (synapse) through NGF secretion, thus allowing efficient norepinephrine release (NE). **B** In cardiac ischemic condition, “activated” fibroblasts (myofibroblasts) secrete high levels of NGF most likely associated with sympathetic hyperinnervation of the infarcted area. However, the absence of cardiomyocytes within the infarcted area results in defective NE secretion of sympathetic nerves.

The present findings show that resident cardiac fibroblasts are the main source of immature pro-NGF and mature NGF in healthy heart tissue and that fibroblasts (but also “pathological” myofibroblasts) promote PC12 differentiation into neurone-like cells most likely consistent with a prominent role of mature NGF. Indeed, mature NGF was shown to be a potent neuronal differentiating and survival factor while, by opposition, proNGF was most likely associated with pro-apoptotic activity [40], [41]. Although we demonstrated that isolated cardiac fibroblasts are naturally able to produce high level of immature pro-NGF and to process it into mature pro-neuronal-differentiating NGF, this phenomenon could be even amplified by other neighboured cardiac cells like cardiomyocytes through proteases secretion. In fact, a number of studies encompassed the role of multiple proteases to cleave proNGF into mature NGF [41], [42]. Moreover, in the context of the neuro-cardiac synapse, despite fibroblasts are dispensable for the PC12-cardiomyocyte synapse functionality since they do not modify NE secretion, our results show that their presence in proximity of the neuro-cardiac synapse seems to play an essential role in the stability and the maintenance of the architectural organization of the synapse by continuously secreting NGF. Thus, overall, our findings strongly suggest that the neurocardiac synapse will be only functional under the control of a triptych and tight cooperation between cardiomyocytes, nerve endings and fibroblasts (Figure 6).

Given that cardiac fibroblasts are prone to many modifications over a large range of cardiac pathologies (hypertrophy, dilatation, MI, etc.) in which reparative fibrosis occurs and that cardiac diseases are frequently associated with SNS dysregulation [43], [44], cooperation between activated fibroblasts (myofibroblasts), cardiomyocytes and sympathetic nerves might be altered in pathological situations and underlined SNS dysregulation. In order to evaluate the interaction between myofibroblasts and neurocardiac synapse cell partners, we used a classical model of myocardial infarction characterized by a massive accumulation of myofibroblasts within the ischemic area. In addition to overexpressed both proNGF and mature NGF as previously described [45], isolated myofibroblasts promoted PC12 cell differentiation into neuron-like cells. As we observed in isolated myofibroblasts, NGF expression was significantly increased in the ischemic cardiac tissue correlating with high myofibroblasts density. Thus, NGF overproduction by myofibroblasts but also macrophages [24] might play a more prominent role in the sympathetic hyperinnervation in the scar while cardiomyocytes present only in the peri-infarct area could contribute to the sympathetic hyperinnervation around the scar. However, despite a large and anarchical TH-immunostaining reflecting sympathetic hyperinnervation in the myofibroblast enriched zone, no significant difference in NE tissue content was noticed when compared to normal cardiac tissue, thus suggesting a functional defect of sympathetic nerves in the scar. Although a large amount of studies have previously described cardiac sympathetic hyperinnervation or altered sympathetic function in cardiopathies, only few studies reported both events in a pressure overload-induced myocardial hypertrophy [46], a monocrotaline-induced right ventricle pressure overload [32] or in ischemia-reperfusion myocardial infarction [47]. Similarly to that observed in cardiac hypertrophy with fetal gene reprogramming of cardiomyocytes, such discrepancy could be explained by rejuvenation of cardiac sympathetic nerves as proposed by Kimura *et al* [32] with development of immature nerves expressing PSA-NCAM as we confirmed in this study in our myocardial infarction model. Some studies suggested that sympathetic depressed function could relied on the downregulation of both NE synthesis [32] and NE secretory machinery [19]. These results reveal the absence of functional synapse between sympathetic nerves and myofibroblasts in ischemic ventricles which parallels the absence of functional synapse between cardiac fibroblasts and nerves in physiological conditions that we demonstrated in the first part of this study. Interestingly, the absence of cardiomyocytes within the infarcted area and thus the lack of functional neurocardiac synapse question the role of this hyperinnervation. NGF overexpression is widely observed in cardiac pathologies (MI, diabetes, etc.) and more likely plays a beneficial role in cardiac cells survival and heart function [15], [16]. Similarly, NGF overexpression by myofibroblasts could be part of an automatic sympathetic nerves rescue program by these cells.

Our results about the role of fibroblast and myofibroblasts in NGF secretion in the heart deserve some comparisons with their role in brain. Indeed, in brain, NGF and other neurotrophic factors are normally secreted by neurones but also by non-neuronal cells such as fibroblasts, both sources being necessary for neurone development, synaptic plasticity and finally for the maintenance of functional synaptic networks [48], [49]. By contrast, in the heart where NGF secretion by cardiomyocytes in physiological conditions seems to be anecdotic, fibroblasts play a primary role in cardiac neuronal network. It has been shown in the brain that the dramatic increase of NFG secretion by non-neuronal cells in response to ischemia prevent early neuronal death and promoting neurite outgrowth of damaged neurons [50]. In the heart, similarly to the protective role reported for NGF in brain, most studies highlighted the cardioprotective role of NGF in the periphery of infarct area where cardiomyocytes more than fibroblasts seem to be the major secretors [15]. By opposition, we showed that high NGF secretion by myofibroblasts isolated from the ischemic area is specifically associated with disorganized cardiac sympathetic hyperinnervation. Thus, NGF production within the infarcted area may be deleterious through the establishment of sympathetic hyperinnervation and cardiac tissue disorganization. So, our study provides evidence for a dual and opposite role of NGF in ischemic cardiac tissue and challenges our current vision of cardiac NGF beneficial effect.

In conclusion, this work highlighted for the first time the essential role of the three cell partners – cardiomyocytes, fibroblasts and nerve endings – in the proper functioning of the neurocardiac synapse and shows the key role of fibroblasts in the neurocardiac synapse stability through NGF production.
